# Qualitative Analysis of Cognitive Interviews With School Children: A Web-Based Food Intake Questionnaire

**DOI:** 10.2196/publichealth.5024

**Published:** 2016-11-28

**Authors:** Vanessa Fernandes Davies, Emil Kupek, Patricia Faria Di Pietro, Maria Alice Altenburg de Assis, Francilene GK Vieira, Clarice Perucchi, Rafaella Mafra, Debbe Thompson, Thomas Baranowski

**Affiliations:** ^1^ Public Health Santa Catarina Federal University Florianopolis Brazil; ^2^ Nutrition Department Santa Catarina Federal University Florianopolis Brazil; ^3^ Department of Pediatrics Baylor College of Medicine Houston, TX United States

**Keywords:** dietary assessment, children, computer, questionnaire

## Abstract

**Background:**

The use of computers to administer dietary assessment questionnaires has shown potential, particularly due to the variety of interactive features that can attract and sustain children’s attention. Cognitive interviews can help researchers to gain insights into how children understand and elaborate their response processes in this type of questionnaire.

**Objective:**

To present the cognitive interview results of children who answered the WebCAAFE, a Web-based questionnaire, to obtain an in-depth understanding of children’s response processes.

**Methods:**

Cognitive interviews were conducted with children (using a pretested interview script). Analyses were carried out using thematic analysis within a grounded theory framework of inductive coding.

**Results:**

A total of 40 children participated in the study, and 4 themes were identified: (1) the meaning of words, (2) understanding instructions, (3) ways to resolve possible problems, and (4) suggestions for improving the questionnaire. Most children understood questions that assessed nutritional intake over the past 24 hours, although the structure of the questionnaire designed to facilitate recall of dietary intake was not always fully understood. Younger children (7 and 8 years old) had more difficulty relating the food images to mixed dishes and foods eaten with bread (eg, jam, cheese). Children were able to provide suggestions for improving future versions of the questionnaire.

**Conclusions:**

More attention should be paid to children aged 8 years or below, as they had the greatest difficulty completing the WebCAAFE.

## Introduction

Dietary assessment among children represents a challenge to nutritional epidemiology due to systematic errors (ie, gender, weight, irregular food patterns, social desirability) [1-4]. In addition, errors related to children’s cognitive development can impact their memory of foods they have eaten and their ability to differentiate between dishes and estimate food portions [5,6].

Web-based questionnaires for assessing children’s food intake have been developed as an alternative to traditional dietary recall methods [7-12]. The advantages of Web-based questionnaires include the following: the extensive interactive resources that can be used to increase children’s motivation to complete the questionnaire, as well as to hold their attention; the standardization of questions, allowing for better quality control; the possibility of self-reporting by children, which reduces the cost of selecting and training interviewers; and the possibility of obtaining quick results [13,14]. A common technique in Web-based questionnaires is to use a character (an avatar) to interact with, guide, or assist children with their recall [15].

In Brazil, the System for Monitoring Food Intake and Physical Activity of Schoolchildren—the WebCAAFE—was developed [16] as a Web-based questionnaire that used an avatar in the form of a robot to assist children with their recall. Its purpose was to enable schoolchildren to self-report their food consumption and physical activity (24-hour recall) within their school environment. Formative and usability studies were conducted [17-19], and the WebCAAFE section related to food intake was validated [8]. Direct observation of school lunch as a reference method showed that the WebCAAFE presented similar validity to other Web-based food intake questionnaires [10,20] (43% matches, 29% intrusions, and 28% omissions), and there was greater disagreement in the responses of younger children (<8 years) compared with older children [8].

Since children receive instructions from the avatar to self-report food intake, ideally having no contact with the researcher, 3 important points should be addressed. First, the words and instructions should be clearly understood by those being interviewed. Technical terms that are not part of the children's daily life can be problematic. Second, questions should be designed taking into account the respondents’ cognitive skills and daily activities. Finally, the answer options must be easily understood by the children [21,22]. The WebCAAFE validation studies raised some important issues about the children’s ability to understand and solve problems when answering the questionnaire. Therefore, to respond to the issues raised and improve children’s performance in the WebCAAFE questionnaire in future studies, cognitive interviews were conducted as a follow-up to the validation studies. The researchers also expected to gain some insights into how Brazilian children answered food questionnaires. The majority of research using qualitative methods to improve food intake questionnaires has been conducted in different sociocultural contexts [11,23-26], and cognitive interviews could fill an important gap in Brazil and similar contexts.

Therefore, this study presents the cognitive interview results of children who answered the WebCAAFE, to obtain an in-depth understanding of children’s response processes.

## Methods

### Participants

An intentional sample [27] of children (based on age and gender) from second to fifth grade (approximately 7-11 years old) at a public primary school in Florianopolis, Brazil, was invited to participate in the cognitive interviews. The ages of Brazilian schoolchildren in specific grades were usually as follows: second grade: 7-8 years; third grade: 8-9 years; fourth grade: 9-10 years; fifth grade: 10-11 years. Ten children participated from each grade (total: 40 children, 21 boys). The choice of school where the study would be conducted was based on the existence of a suitable computer room and Internet connection for the interviews to be carried out. The number of children chosen to be interviewed was determined by the principle of saturation of the collected data (ie, saturation of information was the criterion used to determine the end of the qualitative data collection) [27]. The teachers were responsible for choosing the participants based on the probability that their parents would give their consent for their children to participate. A consent form was sent to the parents or guardians to authorize the participation of their children in the study. Children whose parents did not provide consent (n=6) were replaced by others. At the time of the interview, the children’s assent was also required for them to participate. The project was approved by the Ethics Committee on Human Research at the Federal University of Santa Catarina (UFSC) (Protocol 2250/11).

### The WebCAAFE Questionnaire

The WebCAAFE questionnaire was a single-day recall procedure from the previous day divided into 3 sections: (1) registration form, (2) diet, and (3) physical activity [19]. The registration section referred to information about respondents, such as their name, mother’s name, sex, weight, height, age, date of birth, and study period. The physical activity section was divided into the 3 parts of the day (morning, afternoon, and evening). Children could choose sedentary and physical activity icons from 32 options.

**Figure 1:**
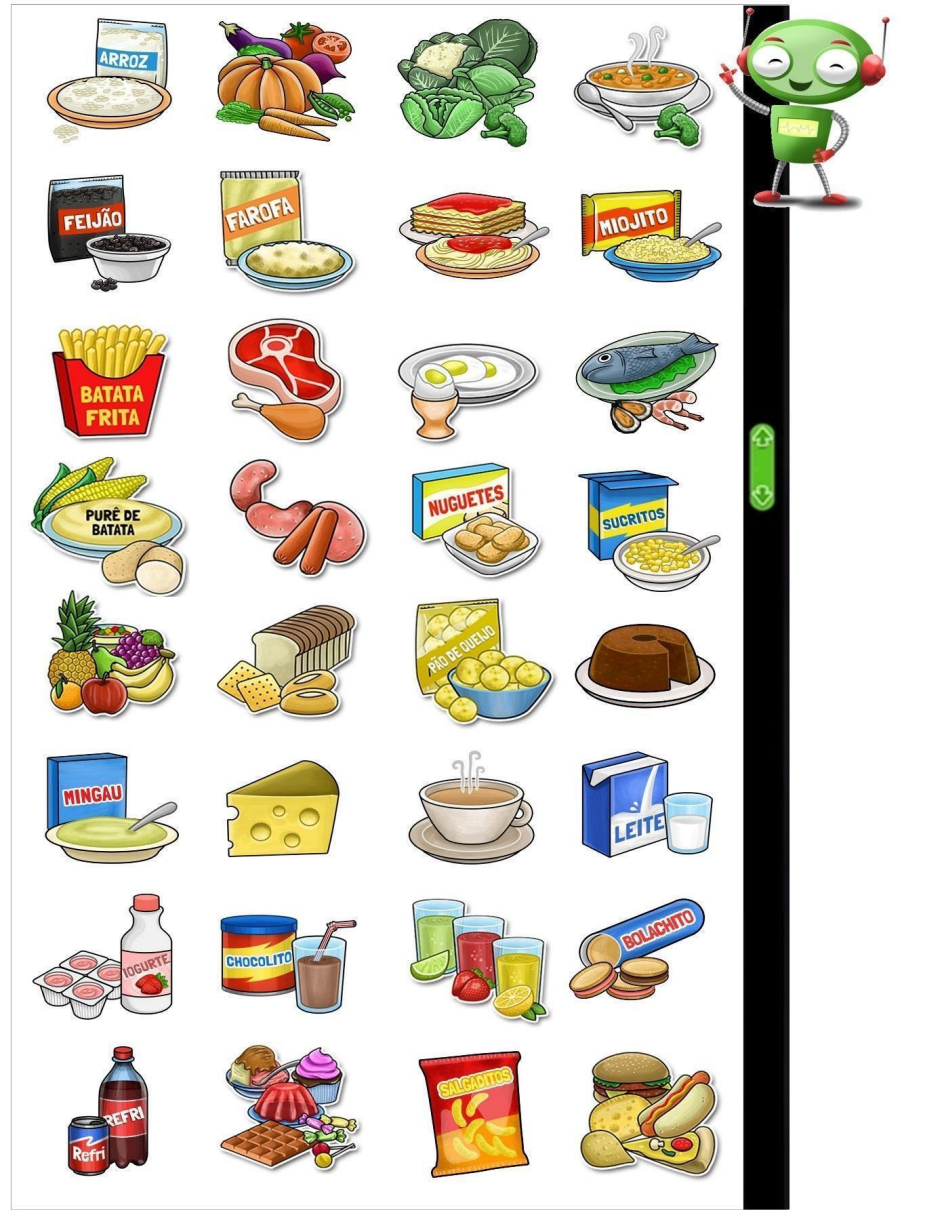
Screenshot of the WebCAAFE questionnaire food options from which the children chose what they consumed the previous day.

**Figure 2:**
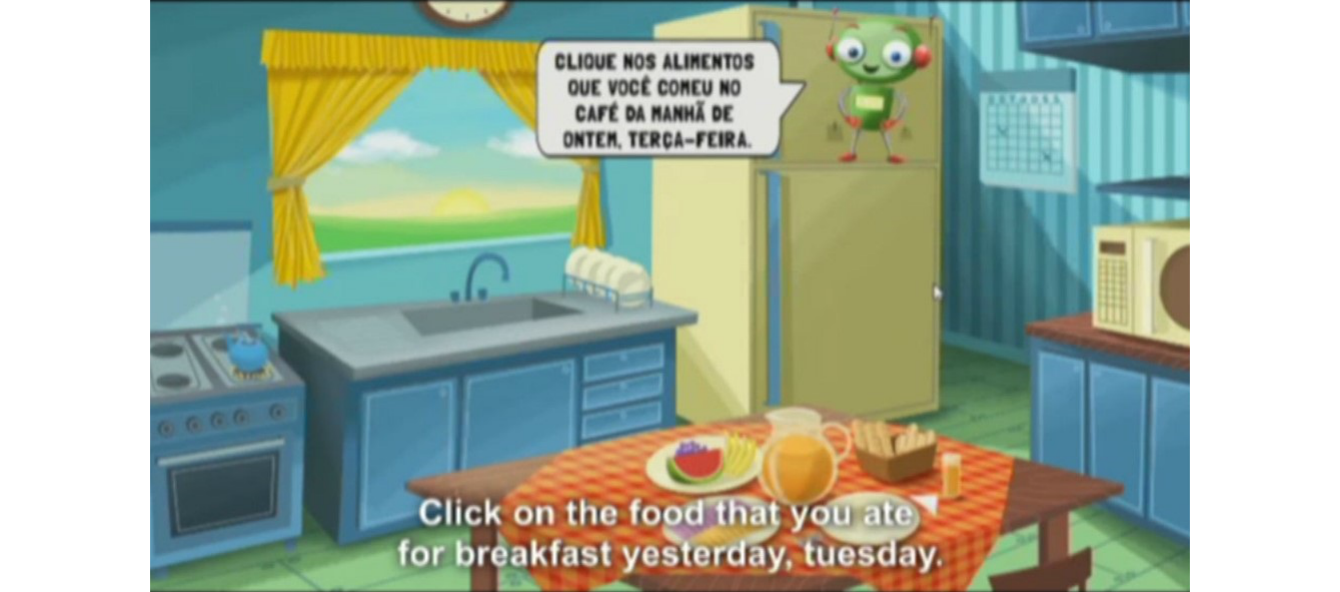
Screenshot of the WebCAAFE showing the instructions given by the avatar for the children to report their food consumption in the previous 24 hours.

**Figure 3:**
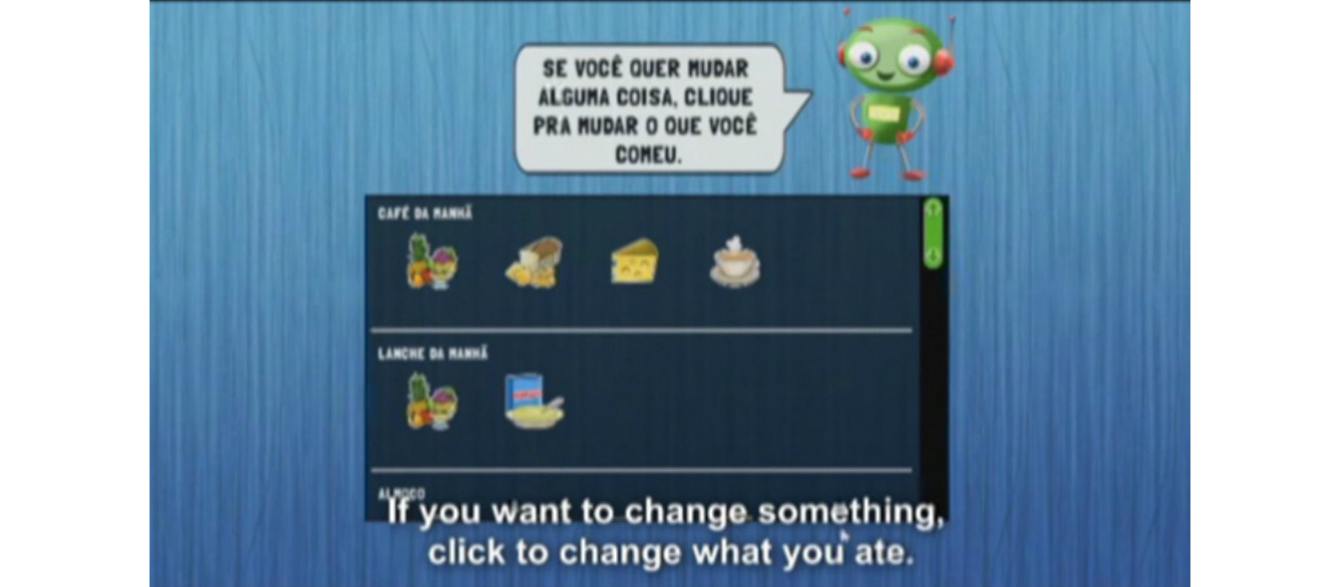
Screenshot of the WebCAAFE showing the instructions given by the avatar for the children to review the foods previously selected.

The food intake section, the objective of this study, was divided into 6 eating occasions (breakfast, morning snack, lunch, afternoon snack, dinner, and evening snack). For each meal, 32 pictures of foods or beverages were displayed on the computer screen so that the child could make their selections (Figure 1). The foods groups, including healthy and unhealthy items, were chosen taking into account the food patterns of children in this age group (reported by schoolchildren in the 7-day food diaries), foods offered in school meals, and foods recommended in the Brazilian food guidelines [19]. A robot avatar guided the child during the interview, explaining the concept of each meal and the time of day at which it was normally consumed, and reinforced the importance of only reporting food intake from the day before (Figure 2). On completing the answers, the child was offered the chance to review the selected foods and make any necessary changes (Figure 3). After completing the 6 eating events, questions about school meals were asked on different screens: (1) “Did you have a school meal yesterday?” (yes/no); (2) if yes, “Which of the foods you selected did you eat during the school meal?” The WebCAAFE system has a database of 350 icons of foods and beverages, as well as 50 icons of different physical activities or sedentary behaviors. This database allows for the selection of 32 icons of foods/beverages and 32 icons of physical activities or sedentary behaviors.

### Data Collection

The cognitive interviews were conducted individually between August and September 2014 in the computer room at the participants’ school. The average session took 60 minutes. The interviews were conducted by one of the authors (VFD) and undergraduate nutrition students (n=6) who had been trained in qualitative techniques and cognitive interviews (4 training sessions, mean duration of sessions: 90 minutes). Before collecting data, interviewers practiced the cognitive interviews with 6 children of similar age to those in the study. Cognitive interviews were conducted at the same time as children were answering the WebCAAFE Web-based questionnaire. The interview script is presented in the Table 1. To ensure consistency in the data collection, the interview script was developed according to the following structure: main question; question to expand and clarify the child's response; and follow-up question (to link the children’s answers to possible suggestions for improving the WebCAAFE). The script was piloted in a test with 10 children from second grade (5 boys and 5 girls) to rephrase or refine the questions in the cognitive interview.

**Table 1:** Examples of questions on the WebCAAFE questionnaire and guides for conducting main questions, probes, and follow-up questions during cognitive interviews sessions with schoolchildren.

WebCAAFE question asked by the Avatar	Aim of the question	Main question	Prompts	Follow-up question
Breakfast is the first meal of the day	To know whether the child understands the word meal and know the meals of the day	Can you tell me in your own words what the Avatar is saying to you ?	What does the word meal mean to you? Can you give me one example? Is breakfast an example of a meal? Can you give other examples of meals? - For example, you said that breakfast is a meal. When do you think you have it (that meal)? Where do you have it?	Do you think we should change any words so the children can understand better? -For example, you said that you didn’t understand breakfast. How could you make it better? - Which other ways do you suggest? - Which word or words do you suggest to improve this question?
Breakfast is the first meal of the day after we wake up. Click on foods that you ate for breakfast yesterday. Remember, if you did not eat anything, click on the ‘nothing’ button	To know if the children understand instructions given by the Avatar. In other words, they should know that they need to answer about foods/drinks they consumed for breakfast yesterday; if they did not eat anything or if they do not remember, the correct answer should be ‘nothing’*.	Can you tell me in your own words what the Avatar is saying to you?	How do you choose something that you ate? If you didn’t eat anything, but drank juice, what should you do? Is the Avatar asking about today or another day?	
Breakfast is the first meal of the day after we wake up. Click on foods that you ate for breakfast yesterday. Remember, if you did not eat anything, click on the ‘nothing’ button	To understand how children’s response processes work	Can you tell me why you chose those foods/drinks?	-Did you find everything that you were looking for? -Can you give me an example of how you would answer if you had a cheese sandwich/tuna pie/pasta with bolognese sauce/a sandwich with chicken paté/an omelet/ bread and jam/vegetable pie -Let’s say that you ate something, but the image of that food is not on screen. What would you do? -If you can’t remember what you ate yesterday, what should you do?	
Now, let’s talk about the morning snack**. This is the meal that you eat after breakfast and before lunch. This would be the meal that you normally have at school. Click on foods that you ate for the morning snack yesterday. Remember, if you did not eat anything, click on the ‘nothing’ button	To understand how children answer questions about foods that they consumed at school	Can you tell me why you chose those foods/drinks?	-Let’s say that you brought food from home. How would you answer that question? - How would you answer this question if you did not come to school yesterday? - How would you answer if you came to school yesterday, but did not eat anything?	
If you want to change anything, click on the button to change what you ate/drank. If you don’t want to change anything, click on ‘continue’	To verify if the children understand instructions. In other words, they should know that the foods presented on the screen are the ones that they chose previously, and at this time they should decide if they want to change any of the answers they gave related to food consumption	Can you tell me in your own words what the Avatar is saying to you ?	Do you recognize these foods/drinks? -Take a look at the screen. Are these the foods and drinks that you chose earlier? (ask this question only if the children do not recognize the foods on the screen) - What do you think you should do now? - What do you think you should do if you want to change something? - What should you do if you don’t want to change anything? - What do you think you should do if you just remembered a food or drink that you had yesterday, but that you forgot to choose?	

* This question should be asked ​​only after the child chooses the foods consumed, or if the child decided to click on the ‘nothing’ button.

** If the child goes to school in the afternoon, this question should be asked about the afternoon snack.

### Data Analyses

All the interviews were audio recorded and transcribed verbatim by the interviewers. The transcripts were compared with the respective audio recordings by 2 researchers (VFD and MRI) to verify accuracy. The same researchers coded the interviews individually (manually), using a thematic matrix technique, within a grounded theory framework of inductive coding [28]. The thematic matrix method included the following steps: rereading the transcripts to identify themes for the organization of data; indexing the themes within the transcripts; and removing data from the transcripts and transferring them to a matrix theme (each theme relating to quotes from the participants). The matrix included the main ideas and perceptions of the individual related to the theme. On completing the coding, analyses were compared, discrepancies were discussed, and a consensus was reached.

## Results

### Principal Findings 

A total of 40 children participated in the interviews (mean age: second grade, 7.8 years; third grade, 8.9 years; fourth grade, 9.4 years; fifth grade, 10.8 years; 21 boys). Four themes were identified: (1) the meaning of words, (2) understanding instructions, (3) ways to resolve possible problems, and (4) suggestions for improving the questionnaire. Throughout this study, the children’s comments were identified by the letter C (short for the word “child”) and their school grade to preserve the participants’ confidentiality.

#### Theme 1: The Meaning of Words

The words specifically investigated were “meal” and “school meal.” The aim of investigating the term “meal” was to assess the child’s recognition of eating occasions (meals or snacks) during the day, the time at which they were consumed, and the types of food consumed at each meal. The term “school meal” was tested to ascertain whether the children associated these words with meals consumed at school, as the WebCAAFE also sought to record information about food consumed at school.

Most children did not know the meaning of the word “meal.” Regardless of age, when asked about this word children cited examples of foods or said the meaning was the same as “food.” When subsequent questions (probes) were asked to expand the children’s answers (the interviewer asked specifically about each meal, for example: “Do you know what breakfast is?”), children cited examples of foods normally consumed in those meals (eg, coffee and bread for breakfast, rice and beans for lunch). Children also correctly identified the time of day and where breakfast, lunch, and dinner were eaten. Eating between meals, such as morning, afternoon, or evening snacks, was recognized only when the snack was consumed at school.

Is there an evening snack as well? I didn’t even know we had stuff like that.

C, second grade

I’ve never used the words evening snack...afternoon snack...morning snack...I don’t say anything like that...I just say dinner and lunch...and breakfast...when I wake up early.

C, fourth grade

Children aren’t used to having some meals and they would get confused when the Avatar asks about afternoon snack, morning snack and evening snack

C, fourth grade

The word “school meal” was understood by the children to mean all food prepared by the kitchen staff.

#### Theme 2: Understanding Instructions

This category included children’s responses regarding their comprehension of the instructions given by the avatar to answer questions about their food consumption and review the foods that they had previously chosen.

In general, children understood the directions and what they were being asked to do. Few children did not know the period (yesterday) about which they should report.

The children who did not usually eat a morning or evening snack were often confused by the question about food consumption for these eating occasions.

“I never knew there was one...” (Comment from a child when asked by the avatar to report food intake in the morning snack)

C, third grade

The snack consumed at school was an important reference for the child to know which foods they had consumed at what time. When asked about this meal, the children seemed to understand that they should record any food consumed at school, whether in school meals, bought in the school canteen, or brought from home. The most frequent doubts were about situations in which children were not at school at the time when the school snack was consumed and did not know whether they should still answer what they had consumed at home or elsewhere.

One instruction given by the avatar that appeared to be understood by most children was when they were asked to check their answers and change anything they thought necessary.

#### Theme 3: Ways to Resolve Possible Problems

This category included the ways in which the children answered the questionnaire when they did not remember what they had eaten the previous day or when they could not find the food images that represented their food intake. This category also recorded how the children answered the questions relating to eating occasions that were not part of their daily routine or that they did not know, such as the morning and evening snacks.

Seven-year-old children who did not remember what they had eaten the day before selected foods at random:

“I'll pretend I ate it.” (Child clicked randomly on various foods)

C, third grade

Older children (9 years old and above) who did not remember what they had consumed selected the “nothing” button. However, there were some situations in which this age group did not remember what they had consumed and they considered clicking on foods that were usually eaten.

I don’t remember what I ate, so I thought about choosing soup, which is what I have when it’s cold...but then I thought again and decided to tell the truth, which is: I don’t remember what I had.

C, fourth grade

Younger children, when they could not find the picture of the food they were looking for, chose other foods to compensate. “Then I'll put juice, it’s easier. Juice is the same as water, it’s made of water.”

C, second grade

For the group of children aged 7 and 8 years, another possibility to compensate for the lack of an image would be to ask for an adult’s help. In some interviews, children were heard to comment that they would ask the teacher when they did not know what to do or they could not find the food they had consumed. During the interview, some food images in the WebCAAFE were not recognized by the children, such as chicken nuggets, the pasta group, cheese bread, manioc flour, porridge, and mussels.

To understand children’s response processes, interviewers asked the children to locate foods on the WebCAAFE screen usually found on the school menu. The foods investigated were cheese sandwich, bread with jam, bread with chicken pâté, vegetable pie, tuna pie, and pasta with minced beef. To locate these foods, the children would ideally make an association with the individual components of the dish (eg, pasta with minced beef—the child would select the pasta and the meat separately, or would select the main component (eg, bread with jam, the child would select at least the bread, as there was no image of jam).

The children’s answers to this question varied by age. Among younger children (7 or 8 years old), some chose food at random that was in no way similar to the investigated food. Others associated foods that were markers of healthy eating, such as bread and cheese with the group of pizzas and hamburgers, or pasta and minced beef with noodles and sausages. A few others reported that the food image could not be found in the WebCAAFE. In addition, many children identified the main food on the screen, such as pasta or bread.

“If I’d eaten bread and cheese, I’d just answer bread, and for pasta with minced beef, I’d only answer pasta.”

C, third grade

It seemed that children aged 9 years or above tried to choose the most appropriate image even if in some cases, the nutritional content of the foods was not fully comparable. For example, bread and fish were chosen to represent tuna pie; bread and sweets represented bread with jam; bread and fruit represented bread with jam; the vegetables group represented vegetable pie; pasta and vegetables represented vegetable pie. Children in the same age group wanted to provide details of preparation, even adding specific ingredients (eg, onions, sour cream).

“It’s the way my grandma makes omelet, she makes it with onion and carrot” (justifying the answer given for what she had eaten: omelet).

C, fifth grade

With regard to the morning and evening snacks, 2 response patterns were observed: (1) reporting consumption of a particular food even if they had previously said during the interview that they did not know what these snacks were or did not eat at those times and (2) confusion among the children relating the morning snack and the evening snack to breakfast or with the dessert at dinner.

#### Theme 4: Suggestions for Improving the Questionnaire

This category included the suggestions made by the children for improving the WebCAAFE. Although the WebCAAFE showed the name of the food when the child passed the cursor over the image on the screen, this detail was not always noticed by the children. For situations in which the food images were not recognized, some children suggested subtitles:

“You could add something below the images to describe what the foods are, for example, to write coffee below the coffee image, soup below the soup image…”

C, fifth grade

Should there not be an image of a food consumed, children suggested writing the name of the food in the space provided: “It would also be nice to write...I would like to write....the foods, if there wasn’t a food you could write it down.”

C, fourth grade

Another suggestion was the inclusion of more food illustrations:

At least put water. You can put more things, if there aren’t many things we won’t know what we ate or what we didn’t eat. Then, when we remember what we ate, we need to have more choices, because only these things, we hardly eat any of them.

C, second grade

Regarding the understanding of instructions, one child suggested offering the option of listening to the avatar’s instructions again:

“I think you could do that...the child didn’t understand...goes there and clicks on the little robot and he appears again, speaking so the child can understand better.”

C, third grade

## Discussion

### Principal Findings

The main results from the cognitive interviews revealed important insights into how children understood and prepared answers to the avatar’s questions, as well as suggestions for improving the questionnaire. The eating occasions which were most recognized by the children were breakfast, the snack eaten at school, lunch, and dinner. Instructions on how to report food intake were also understood by the majority of children, as well as the instructions to review their selected foods at the end of the questionnaire. Younger children were less able to solve problems and were less consistent in their response processes compared with older children. These results answered questions raised by the WebCAAFE researchers during the validation study, as well as reflected on the possible ways of dealing with children’s difficulties in future applications of the questionnaire.

It has been recommended that Web-based questionnaires to assess children’s food intake follow a guided meal-based approach to help children recall information [11,12,29]. Our interviews, however, showed that for the sample of children participating in this study, eating occasions such as morning and evening snacks were either not part of their usual routine or not known by the children, and may therefore be confused with other meals. This problem was also identified in a formative study with nutritionists for the development of the WebCAAFE [17]. Children who did not consume snacks at these times were induced to respond that they did consume them, thus increasing errors. These results indicated that children needed a more detailed explanation of the types of meals before completing the WebCAAFE. When the questionnaire was administered at schools, researchers followed a written protocol to explain how to answer the questionnaire in the classroom with children, for which a banner (140 × 105 cm) was used to illustrate the foods and meals included in the WebCAAFE questions. A possible solution would be to explain more clearly the precise meaning of these meals and make it clear to the children that if the meal was not eaten, they should not choose any food.

In Brazil, there is the school feeding program as well as laws prohibiting the sale of processed foods or soft drinks in schools [30]. The results from the cognitive interviews showed that the school meal was an important reference for children, both to help them understand what time of day the avatar was asking about and to remember the foods they had consumed. This is considered a positive result as it shows the potential of the WebCAAFE for assessing food consumed in the school environment, thereby serving as an important tool for evaluating nutrition policies.

Similar to the WebCAAFE validation study, children aged 9 years and above performed better than younger children in this study (4). Children aged 7 and 8 years had more difficulty when searching for images of food that reflected their consumption more literally. This information was in accordance with other studies that experienced the same problem with regard to children’s reporting of mixed dishes, different types of preparation (eg, boiled egg or fried egg), or foods that can be added to other foods (such as jam, cheese, butter, or mayonnaise) [6-10,31-33,34]. For younger children answering the WebCAAFE, the presence of an adult (teacher or researcher) to monitor and solve problems during the administration of the questionnaire could minimize errors. Since some children commented that they would ask the teacher when they did not know what to do or could not find the food consumed, adults could use the school menu as a reference to guide the children to answer the questions accurately. For example, if children had consumed vegetable pie, they could be advised to click on the vegetables group as well as on the breads and crackers group. Standardized guidelines could also be developed to specify procedure in the absence of images (ie, when the child consumed something for which there was no image in the WebCAAFE, they should be advised to click on the “nothing” button). Adult assistance for children younger than 8 years reporting food intake was also recommended in other studies [24,25].

A good understanding of the avatar’s instructions was a positive result, confirming the importance of the research conducted during the development of the WebCAAFE, such as the focus group discussions with nutritionists and the questionnaire usability studies [17,19]. However, should there be future studies with the WebCAAFE in other regions of Brazil, it is important to reevaluate this issue, as there are major socioeconomic differences in the country. It may be necessary to train children before answering the questionnaire so they are better able to answer the questions and solve problems that may arise.

Changes in response to children’s suggestions could contribute to further improvements, for example, the use of more familiar words for children (eg, “school snack” instead of “school meal”), the use of more visible subtitles to identify the food images, more common food options (eg, water), and the inclusion of a space to write the foods consumed. Other Web-based food intake assessment questionnaires have used a write-in feature [10,11]. It should be emphasized that the WebCAAFE system has a database of 350 foods from which 32 food icons can be selected to be displayed on the screen, so the food items can be replaced by others depending on the research and objectives. The findings of this study, as well as discussions with specialists in educational psychology and software development, may help to improve the next version of the questionnaire with explanations that take into account the limitations of children’s vocabulary and add the option for children to write a food if it is not included on the screen.

The strengths of this study included conducting a pilot study to modify and refine the interview questions; the use of an interview script to ensure consistency of the collected data; carrying out research in a school similar to those where the questionnaire would be administered; and extensive training of the interviewers. A limitation of this study was conducting the study in one area, which did not allow for generalizations to be made about the children’s cognitive performance when answering questionnaires in other sociocultural contexts. Regional variations in the school menus in Brazil may also lead to other difficulties for children to answer the same questionnaire.

Future studies should evaluate the addition of an adult as a facilitator when children aged 7-8 years are completing the questionnaire, guiding them through the WebCAAFE screens one by one.

### Conclusions

This study obtained similar results to other studies with Web-based tools for the assessment of food consumption. However, this did not alter the relevance of this study, as the cognitive interviews provided not only important insights for improving future versions of the WebCAAFE questionnaire but also general information about Brazilian children answering Web-based food questionnaires, contributing to the research on this issue in a country with little experience of this type of technological innovation.

The questionnaire structure (6 eating occasions per day) was not understood by many children, as some meals were not part of their normal routines or were unknown to them. Choosing images of mixed dishes and foods eaten with bread (eg, jam) were complex tasks that demanded cognitive skills that younger children might not have fully developed. Finally, as indicated in the WebCAAFE validation study, more attention should be paid to children aged 8 years and below, as they had the greatest difficulty completing the WebCAAFE. The assistance of an adult when younger children are completing the questionnaire could be an alternative way of improving the accuracy of the answers.
